# Expert consensus on peri-implant keratinized mucosa augmentation at second-stage surgery

**DOI:** 10.1038/s41368-025-00379-3

**Published:** 2025-06-19

**Authors:** Shiwen Zhang, Rui Sheng, Zhen Fan, Fang Wang, Ping Di, Junyu Shi, Duohong Zou, Dehua Li, Yufeng Zhang, Zhuofan Chen, Guoli Yang, Wei Geng, Lin Wang, Jian Zhang, Yuanding Huang, Baohong Zhao, Chunbo Tang, Dong Wu, Shulan Xu, Cheng Yang, Yongbin Mou, Jiacai He, Xingmei Yang, Zhen Tan, Xiaoxiao Cai, Jiang Chen, Hongchang Lai, Zuolin Wang, Quan Yuan

**Affiliations:** 1 State Key Laboratory of Oral Diseases & National Center for Stomatology & National Clinical Research Center for Oral Diseases & West China Hospital of Stomatology, Sichuan University, Chengdu, China; 2 Shanghai Engineering Research Center of Tooth Restoration and Regeneration & Tongji Research Institute of Stomatology & Department of implantology, Shanghai Tongji Stomatological Hospital and Dental School, Tongji University, Shanghai, China; 3 Department of Implantology & National Center for Stomatology & National Clinical Research Center for Oral Diseases & National Engineering Research Center of Oral Biomaterials and Digital Medical Devices, Peking University School and Hospital of Stomatology, Beijing, China; 4 Department of Oral and Maxillofacial Implantology, Shanghai Ninth People’s Hospital, Shanghai Jiao Tong University School of Medicine, Shanghai, China; 5 Department of Oral Surgery, Shanghai Ninth People’s Hospital College of Stomatology, Shanghai Jiao Tong University School of Medicine, Shanghai, China; 6 State Key Laboratory of Oral & Maxillofacial Reconstruction and Regeneration, National Clinical Research Center for Oral Diseases, Shaanxi Engineering Research Center for Dental Materials and Advanced Manufacture, Department of Oral Implants, School of Stomatology, The Fourth Military Medical University, Xi’an, China; 7 Department of Oral Implantology, University of Wuhan, Wuhan, China; 8 Hospital of Stomatology, Guanghua School of Stomatology and Guangdong Provincial Key Laboratory of Stomatology, Sun Yat-sen University, Guangzhou, China; 9 Stomatology Hospital, School of Stomatology, Zhejiang University School of Medicine Zhejiang Provincial Clinical Research Center for Oral Diseases, Key Laboratory of Oral Biomedical Research of Zhejiang Province, Cancer Center of Zhejiang University, Engineering Research Center of Oral Biomaterials and Devices of Zhejiang Province, Hangzhou, China; 10 Department of Dental Implant Center, Beijing Stomatological Hospital, School of Stomatology, Capital Medical University, Beijing, China; 11 Department of Oral Implantology, School and Hospital of Stomatology, Jilin University, Changchun, China; 12 Department of Oral Implantology, Tianjin Stomatological Hospital, School of Medicine, Nankai University & Tianjin Key Laboratory of Oral and Maxillofacial Function Reconstruction, Tianjin, China; 13 Department of implantology, Stomatological Hospital of Chongqing Medical University, Chongqing, China; 14 Department of Oral Implantology, School and Hospital of Stomatology, Key Laboratory of Oral Diseases of Liaoning province, China Medical University, Shenyang, China; 15 Jiangsu Key Laboratory of Oral Diseases, Nanjing Medical University; Jiangsu Province Engineering Research Center of Stomatological Translational Medicine, China; Department of Dental Implantology, The Affiliated Stomatological Hospital of Nanjing Medical University, Nanjing, China; 16 Fujian Key Laboratory of Oral Diseases & Fujian Provincial Engineering Research Center of Oral Biomaterial & Stomatological Key Laboratory of Fujian College and University, School and Hospital of Stomatology, Fujian Medical University, Fuzhou, China; 17 Center of Oral Implantology, Stomatological Hospital, School of Stomatology, Southern Medical University, Guangzhou, China; 18 Department of Stomatology, Union Hospital, Tongji Medical College, Huazhong University of Science and Technology, Hubei Province Key Laboratory of Oral and Maxillofacial Development and Regeneration, Wuhan, China; 19 Nanjing Stomatological Hospital, Affiliated Hospital of Medical School, Nanjing University, Nanjing, China; 20 College & Hospital of Stomatology, Anhui Medical University, Key Lab. of Oral Diseases Research of Anhui Province, Hefei, China

**Keywords:** Dental implants, Oral diseases

## Abstract

Peri-implant keratinized mucosa (PIKM) augmentation refers to surgical procedures aimed at increasing the width of PIKM. Consensus reports emphasize the necessity of maintaining a minimum width of PIKM to ensure long-term peri-implant health. Currently, several surgical techniques have been validated for their effectiveness in increasing PIKM. However, the selection and application of PIKM augmentation methods may present challenges for dental practitioners due to heterogeneity in surgical techniques, variations in clinical scenarios, and anatomical differences. Therefore, clear guidelines and considerations for PIKM augmentation are needed. This expert consensus focuses on the commonly employed surgical techniques for PIKM augmentation and the factors influencing their selection at second-stage surgery. It aims to establish a standardized framework for assessing, planning, and executing PIKM augmentation procedures, with the goal of offering evidence-based guidance to enhance the predictability and success of PIKM augmentation.

## Introduction

The long-term success of an implant relies on both osseointegration and the condition of the soft tissue surrounding the implant.^1^ Adequate and healthy mucointegration and sealing around the implant are crucial for isolating the alveolar bone from the oral environment and bacterial aggression, thereby ensuring the stability of implant osseointegration.^2^ *The ITI treatment guide series* emphasizes the importance of the peri-implant soft tissue, particularly in patients with conditions such as soreness during oral hygiene procedures, thin mucosa associated with bone regenerative procedures, suboptimal plaque control, peri-implant recession or shallow vestibule.^3^

Unlike periodontal soft tissue, peri-implant soft tissue is composed of the peri-implant connective tissue, internal sulcular/junctional epithelium, and outer surface mucosa.^4^ The peri-implant keratinized mucosa (PIKM) is a stratified squamous epithelium located between the mucogingival junction (MGJ) and the mucosal margin. Typically, the width of PIKM varies among patients and even within different areas of the same patient’s oral cavity. PIKM resists mechanical friction, stabilizes the gingival margin, reduces plaque accumulation, and withstands tension from mucosal muscle fibers.^5–7^ The necessity of a minimum width of PIKM for the long-term maintenance of peri-implant health has been debated for many years. However, recent evidence and consensus reports increasingly indicate that an inadequate width (2 mm or less) of PIKM is associated with a higher prevalence of peri-implantitis, biofilm accumulation, soft-tissue inflammation, mucosal recession, marginal bone loss, and increased patient discomfort.^8^ In 2018, the Osteology Foundation Consensus Meeting concluded that soft tissue augmentation procedures are beneficial for promoting peri-implant health.^9^ A recent consensus report recommends routinely assessing the width of PIKM in patients with implant-supported restorations. While pathological changes in the peri-implant mucosa are associated with insufficient PIKM, its dimensions can be surgically increased using autogenous grafts or soft tissue substitutes with proven efficacy.^10^

PIKM augmentation procedures can be performed before implant placement, during the second-stage surgery, or after the final restoration.^11^ The second-stage surgery is considered crucial for PIKM augmentation as it enables direct soft tissue management and ensures optimal healing prior to prosthetic restoration.^12^ This stage provides an opportunity to assess the existing soft tissue conditions and address any deficiencies in PIKM that may compromise peri-implant health or esthetics. At this stage, the implant has already achieved osseointegration, and the soft tissue environment is more stable compared to the initial placement or post-restoration phases, which reduces surgical risks and enhances the predictability of augmentation outcomes.^13^ Furthermore, the second-stage surgery is typically the final surgical procedure in implant rehabilitation. Addressing soft tissue management at this stage is conducive to maintaining the width of KM and reducing the need for additional procedures and patient visits. Additionally, assessing and correcting PIKM deficiencies before prosthetic restoration minimizes the need for future surgical interventions, which otherwise could complicate treatment by requiring modifications to the prosthesis, increasing treatment time and complexity, and reducing predictability.^13,14^ From the patient’s perspective, performing PIKM augmentation at the second-stage surgery is typically integrated into the overall treatment plan, without causing additional trauma or extending treatment time, making it more acceptable.

Various surgical procedures have been developed to preserve and/or reconstruct PIKM, ensuring flexibility in addressing peri-implant soft tissue deficiencies. These techniques include, but are not limited to, apically repositioned flap (ARF), autogenous free gingival graft (FGG), and allogenic or xenogenic soft tissue graft.^15–21^ Although these techniques have been widely used, their clinical indications remain unclear. Furthermore, the advantages, disadvantages, and technical considerations vary among the different procedures, highlighting the requirement for standardized decision protocols to guide their application. Based on current research, this consensus summarizes the advantages and disadvantages of various commonly used techniques for PIKM augmentation, clarifies their indications and technical nuances. The objective is to establish a standardized framework for the assessment, decision-making, and execution of PIKM augmentation procedures, especially during second-stage surgery. This framework will provide clinicians with practical guidance to improve their success rates and ensure the long-term success of implant therapy.

## Peri-implant keratinized mucosa augmentation surgery

This section provides an overview of common surgical techniques for augmenting the width of PIKM, including ARF, FGG, Strip free gingival graft (SFGG), and the use of soft tissue substitutes. It discusses the underlying concepts, key procedural steps, as well as the advantages and limitations of each method. Each technique offers benefits, and the choice of procedure should be tailored to the individual clinical scenario to achieve optimal outcomes (Table 1).

**Table 1:** Advantages and disadvantages of different PIKM Augmentation Surgeries

Surgery	Advantage	Disadvantage
ARF	- No need for a second surgical site. - Good color-match. - Optimal for posterior maxilla.	- Application restricted by remaining width of KM in the mandible.
FGG	- Predictable and reliable increase in KM width. - Long-term stability. - Application not limited by remaining width of KM.	- Donor site morbidity. - Color mismatch. - Limited by donor site area.
SFGG	- Hardly limited by donor site area. - Usable for large-scale augmentations - Good color-match compared to FGG. - Less donor site trauma than FGG.	- Technique sensitive. - Time-consuming.
Soft Tissue Substitutes	- Avoids donor site complications. - Good color-match compared to FGG. - Usable for large-scale augmentations. - Shorter surgical duration.	- High cost. - Higher contraction rate.

### Apically repositioned flap

ARF was the first surgical technique developed to increase keratinized gingiva, initially introduced in the 1950s to enhance the attached gingiva for removable prostheses.^22,23^ Over time, the modified ARF (MARF) technique was introduced to extend the dimensions of keratinized mucosa (KM) by apically displacing a partial-thickness flap while preserving the coronal keratinized tissue, effectively minimizing recession during healing.^24–26^ Subsequently, scholars successfully applied this technique in the management of inadequate PIKM. Studies have shown that ARF results in an average KM width increase of 1.15 mm at 12 months postoperatively, with a shrinkage rate of approximately 40%–70%.^27,28^

ARF for dental implant is often performed using a horizontal and bilateral vertical split incision that extends beyond the mucogingival margin, facilitating the apical position of the flap. All coronal incision lines must be located within the KM, then a narrow band of KM reflected with the partial thickness flap is apically displaced and sutured using horizontal mattress suture. This narrow band of KM served as an apical barrier to the nonkeratinized alveolar mucosa, creating a recipient bed with KM on both the apical and coronal sides. Consequently, cells from the surrounding keratinized tissue can migrate into the exposed periosteal area and form KM. Unlike the “strip” technique, which requires a 2 to 3 mm graft width (discussed later), the coronal portion of the flap in ARF provides a sufficient band of KM to ensure the healing process. This may be attributed to the better blood supply provided by the pedicled KM compared with the free gingival strip.

The favorable advantage of ARF technique is that it does not require a second surgical site, which results in better patient acceptance, and it produces better color-match predictability.^29^ However, its therapeutic effect depends on the quality of the surrounding mucosa. Therefore, ARF is highly recommended for the posterior maxillary region, where there is ample KM available on the palatal aspect. By making a horizontal incision approximately 5 mm palatal to the MGJ and two vertical incisions crossing the MGJ, a split-thickness flap is then raised with a very wide band (5 mm) of KM in the coronal portion. The flap is mobilized through deep and superficial split-thickness incisions to allow for apical repositioning. Finally, the flap is sutured to the periosteum in the apical position using horizontal mattress sutures, while simple interrupted sutures are used to secure the remaining incision lines. This technique performed in the posterior maxilla significantly increases the width as well as the thickness of keratinized tissues on the buccal side of the implant. However, it does not improve the height of peri-implant papillae or the palatal thickness of KM due to the secondary healing of the palatal area. To address these limitations, several modified methods have been reported and have shown very promising results.^30–32^ Although ARF provides better tissue color match, it is not suggested to use it in the anterior aesthetic zone due to the presence of palatal rugae.

When this technique is applied to the mandible, at least 3–4 mm width of keratinized gingiva is required. Unlike the maxilla, the mandible does not have a significant amount of KM available on the lingual side. Therefore, the horizontal incision should ensure that approximately 2 mm of KM remains on the lingual side, with the rest repositioned apically to allow the exposed buccal periosteum to self-heal and form new KM. This differs from the maxilla, where the exposed palatal periosteum contributes to the formation of new KM. In addition, the soft tissues in the mandible are often much thinner than the palatal fibromucosa, making it difficult to achieve a significant increase in thickness, which may lead to shrinkage of the widened KM. The shallow vestibule in the mandible also makes it more challenging to anchor the flap, which is more likely to retract due to muscle pulling.

### Free gingival graft

While Bjorn introduced the concept of FGG in 1963 for teeth,^33^ its application around implants was progressively validated in the 1980s and 1990s, particularly for addressing peri-implant issues associated with insufficient PIKM.^34^ Early studies highlighted the importance of KM in maintaining peri-implant health and stability. For instance, Berglundh et al. emphasized the role of soft tissue barriers around implants in protecting the underlying structures and mitigating peri-implant inflammation.^35^ Similarly, research by Simons et al. reported that FGG effectively addresses peri-implant soft tissue complications by increasing the width of keratinized tissue and stabilizing peri-implant soft tissues, thereby improving the long-term prognosis of implant-supported restorations.^36^ Clinical systematic reviews involving large patient cohorts have further confirmed that FGG not only increases the width of PIKM but also reduces peri-implant tissue inflammation and enhances soft tissue stability.^37^

FGG is widely recognized as the gold standard for PIKM augmentation due to its predictability, long-term stability, and versatility across a wide range of clinical applications. Clinical studies have demonstrated that FGG achieve a 93% success rate in increasing the zone of PIKM, with a mean gain of (3.73 ± 1.93) mm, highlighting its reliability and predictability in PIKM augmentation.^38^ This performance surpasses that of other techniques, such as ARF, SFGG, and alternative materials. Despite the initial shrinkage of 20%–40% within the first three months due to primary and secondary contraction, the remaining tissue exhibits superior stability over time.^39^ Thicker FGG (1.5-2 mm), characterized by its robust lamina propria, further minimizes shrinkage and provides enhanced resistance to mechanical forces.^40,41^

Compared to ARF, FGG offers a significant advantage in cases with insufficient residual width of KM. While ARF is effective in situations with an adequate amount of pre-existing KM, it relies on the redistribution of available KM, which limits its indications.^42^ In contrast, FGG has a wide range of clinical applications, can be employed at different stages of implant surgery and increases both the width and thickness of PIKM in sites with minimal or absent keratinized tissue. However, ARF has its advantages, including reduced surgical morbidity, faster recovery, and superior color matching with adjacent tissues, making it a preferred choice in esthetically sensitive regions where the remaining KM width is adequate for executing the surgery.^28^

Unlike ARF, where the residual KM is redistributed, the FGG technique involves making a horizontal incision at the MGJ along the length of the recipient area and extending to adjacent teeth. This approach allows the FGG to be utilized even in cases where the residual width of KM is insufficient for the classical ARF procedure, significantly expanding its indications for KM augmentation. The horizontal incision is connected to two vertical incisions at 90° or slightly divergent toward mucosa. A split-thickness dissection is performed to create a stable recipient periosteal bed. It is important to completely dissect all the elastic and muscular fibers to prevent the formation of movable keratinized tissue. The partial thickness flap is recommended to be fixed at the apical position with “T-mattress” sutures, which combine vertical and horizontal components or external horizontal mattress sutures to prevent coronal displacement.^43^ During suturing process, inverting the non-keratinized mucosa at the edge of the partial-thickness flap is recommended, as it helps prevent the coronal migration of non-keratinized mucosa during healing.

Once the recipient area is prepared, the donor site is selected. As a standard practice, FGG tissue is harvested from the palate between first molar and first premolar on the same side as the recipient side to reduce discomfort and preserve functionality after the procedure. The width of the graft is typically limited to 8–10 mm below the bottom of the gingival sulcus to minimize the risk of injuring the palatine artery.^44^ The graft should be ≥1 mm thick.^41^ Alternative donor sites include other edentulous ridges, the tuberosity area, and the posterior maxillary buccal ridge when sufficient keratinized tissue is present. The latter is particularly favorable for achieving better tissue blending and aesthetic outcomes.^45^ The obtained gingival grafts should be carefully debrided of fat and glandular tissue, and shaped to precisely conform to the recipient area.^46,47^

The graft is secured to the periosteum around its margins using interrupted sutures and is firmly anchored to the periosteum of the recipient site with coronapical cross-mattress sutures. This procedure ensures tight contact with the periosteal vascular bed, promoting capillary anastomosis and successful graft integration.

While highly effective, FGG is associated with several disadvantages that must be considered during treatment planning. One of the primary drawbacks is donor site morbidity. Harvesting tissue from the palate often causes significant postoperative discomfort, including pain, bleeding, and delayed wound healing. Studies have reported that complications at the donor site, such as prolonged sensitivity and impaired function that can negatively impact patient satisfaction.^48^ Additionally, the healing at the donor site may take weeks, during which patients may experience difficulty eating and speaking if the wound is not properly protected.^49^

Another limitation of FGG is the potential for esthetic issues, particularly in anterior regions where color mismatch can be problematic. FGG typically exhibits a lighter, pinker hue compared to the surrounding tissues, making the grafted area appear conspicuous. This is due to the inherent properties of the harvested keratinized tissue, which does not always integrate seamlessly in terms of color and texture with adjacent mucosa.^50,51^ These esthetic limitations make FGG less desirable for high-visibility regions where patient satisfaction heavily hinges on appearance.

FGG is also constrained by the availability of donor tissue. In cases requiring extensive augmentation, the donor site may not provide sufficient tissue to meet clinical needs, necessitating the use of alternative techniques or materials. The palatal tissue commonly used for FGG may not always offer the quantity or quality required, particularly in patients with pre-existing palatal conditions or limited donor areas.^52–54^ These constraints can limit the applicability of FGG in more complex cases.

FGG also carries a risk of complications at the recipient site, such as graft necrosis or inadequate integration. Thick grafts, while providing better long-term stability, may impede vascularization and increase the likelihood of necrosis if not properly adapted to the periosteal bed.^55^ Ensuring close contact between the graft and recipient bed is critical. Certain technical errors, such as improper suturing techniques or discrepancies in the dimensions of the FGG, may significantly increase the likelihood of graft failure or suboptimal results.^56^ These challenges underscore the need for precise surgical planning and execution to maximize the benefits of FGG while addressing its inherent limitations.

Some other autologous tissue grafting procedures are also widely used for soft tissue management, including de-epithelialized free gingival graft (commonly referred to as connective tissue graft, CTG) and palatal roll envelope technique.^57^ CTG is one of the most commonly used soft tissue grafting techniques, primarily employed to increase soft tissue thickness and improve aesthetics.^58^ The procedure involves harvesting connective tissue from the palate or maxillary tuberosity, removing the epithelial layer, and grafting it to the recipient site, followed by coverage and healing. Studies have shown that CTG can effectively enhance soft tissue thickness, improve gingival recession, and provide a certain degree of long-term keratinized mucosa stability.^59,60^ The palatal roll envelope technique, on the other hand, involves creating a pedicled flap from the palate, which is then rotated or slid into the recipient site to enhance soft tissue thickness while maintaining the original blood supply, leading to a higher survival rate.^61^

However, these techniques are primarily indicated for enhancing the thickness, contour, and aesthetics of gingival or peri-implant soft tissue rather than merely expanding the KM width, although they can also increase the width of KM to some extent and improve its long-term stability.^62,63^ Additionally, studies indicate that the KM induction effect of de-epithelialized connective tissue grafts is inconsistent and varies among individuals.^64,65^ Furthermore, to achieve tension-free wound closure, CTG procedures often require coronally advanced flaps or tension-releasing incisions, which may results in a shallower vestibule, potentially reducing the width of keratinized mucosa.^66^ Moreover, CTG-induced KM may also exhibit some degree of color mismatch, lacking a significant esthetic advantage.^67^ Therefore, these techniques are not the primary focus of discussion in this review.

### Strip free gingival graft

The SFGG technique was first introduced by Han et al. in 1993 to decrease the extent of autograft harvesting and minimize trauma to the donor sites.^68^ This technique utilizes thin strips of free gingiva placed parallel to one another and fixed onto the prepared periosteal bed, leaving the exposed periosteum between the graft strips to heal by secondary intention. The strips are typically 1-1.5 mm thick and 2-4 mm wide, spaced evenly to allow secondary epithelialization. Fixation is achieved using sutures, such as horizontal mattress sutures, to ensure stability and prevent displacement during healing. This technique addresses the inherent limitation of FGG by reducing the extensive demand for graft tissue, and decreasing donor site morbidity compared to traditional FGG methods. However, this surgical approach is technically demanding, time consuming, and may still cause discomfort for patients, as multiple strips of autografts are required to be harvested and secured in parallel to cover large portions of the periosteal bed. Additionally, the exposure of the periosteal bed is more likely to cause infection, pain, and delayed healing.^69^

To overcome these challenges, Urban introduced the strip autograft combined with the xenogeneic collagen matrix (XCM) technique, which uses a single strip of gingival tissue placed at the apical portion of the periosteal bed as both a mechanical barrier against alveolar mucosa and muscle tension, and as cell source for desired keratinized gingiva regeneration. Additionally, the XCM, which covers the remaining periosteal bed, serves as a scaffold for epithelium cell ingrowth.^69^ Currently, there is no statistical data addressing the KM width achieved with the SFGG technique alone. However, case series studies on the combination of SFGG with XCM have reported an average KM width of approximately 6 mm at 1 year postoperatively, with a shrinkage rate of 43%.^69,70^ A recent single-arm clinical trial found that the average KM width at 6 months postoperatively was (3.3 ± 1.6) mm for SFGG, compared to (4.6 ± 1.6) mm for FGG. Although the difference was not statistically significant, the numerical difference of over 1 mm may still influence surgical decision-making.^19,71^ A histologic study demonstrated that the expression levels of newformed keratin and collagen were similar to reference samples of palatal autogenous grafts, indicating that the combination of SFGG and XCM provides physiologically normal KM. This technique reduced the donor site morbidity and achieved a better color match compared to FGG.

However, it is still observed that the regenerated KM derived from the palatal strip often differs in color from the adjacent lateral tissue and coronal tissue. Recently, a labial strip gingival graft in combination with XCM has been proposed by Urban to achieve a better color match between the regenerated KM and the surrounding tissue.^72^ However, harvesting labial gingival graft requires a sufficient width of keratinized gingiva on the adjacent labial site; otherwise, the risk of gingival recessions may be heightened in cases without adequate KMW.

Overall, the SFGG does reduce post-operative discomfort in the palate donor area and can, to some extent, improve color mismatch issues compared to FGG. However, studies have reported another issue that cannot be ignored: the noticeable difference in thickness between the KM regenerated by apically fixed SFGG and that regenerated by XCM.^71^ In addition, almost all the existing reports on SFGG techniques require the use of soft tissue substitutes, which increases the financial burden on patients. Based on the current evidence, SFGG is suitable for the maxillary anterior region or large-scale soft tissue augmentation, where a significant amount of keratinized gingiva is not available at the donor site. However, caution is still required as this technique still faces issues with incomplete color matching and the inability to achieve an increase in soft tissue thickness.

### Soft tissue substitutes

Although FGG consistently achieves the greatest gain in PIKM width and has a lower contraction rate, it is associated with increased postoperative discomfort. In order to avoid extra wounds in the palatal region, allografts were used as an alternative to autogenous soft tissue for periodontal soft tissue surgery in the late 1980s. Acellular dermal matrix (ADM) is the most commonly reported allograft used to increase the width of keratinized tissue around teeth and implants.^73,74^ ADM is a freeze-dried matrix that is free of epithelium and cellular components with type I and III collagen bundles and elastic fibers as its main components.^73^ The clinical procedure is similar to that of FGG, except that FGG is replaced with an allograft, which serves as a bioactive scaffold allowing epithelial and endothelial cells migration and integration via the vascular channels of the recipient sites.^75^ ADM has demonstrated clinical efficacy in increasing the width of PIKM by (2.59 ± 0.92) mm and offers advantages such as better color matching, reduced surgical time, and improved patient comfort.^76–78^ However, it also has drawbacks, including lower effectiveness in KM augmentation, higher rates of tissue contraction (ADM: 56%-72% vs. FGG: 12%-16%), and delayed healing (by 5-20 days compared to FGG).^18,73,77,79,80^

The recently developed XCM is a pig-derived resorbable collagen membrane with a bilayer structure; the outer layer is dense, aiding in suturing and protecting the wound under open healing conditions, while the inner layer is porous, promoting blood clot stabilization, cell growth, early vascularization, and accelerating soft tissue healing.^17^ Studies have shown that XCM effectively increases the width of keratinized gingiva around implant.^81^ Compared to FGG, the regenerated tissue often exhibits better integration and aesthetic outcomes.^82^ Additionally, XCM significantly reduced patient morbidity by shortening surgical time, as well as decreasing postoperative swelling, bleeding, and pain.^83–85^ However, XCM presents limitations in terms of shrinkage rates (34% - 51%) and lower effectiveness in increasing KM width (XCM: (1.8 ± 1.0) mm vs. FGG: (4.1 ± 1.6) mm) and thickness (XCM: (1.7 ± 0.6) mm vs. FGG: (1.2 ± 0.3) mm).^17,86^

Currently, several alternative soft tissue substitutes are being explored for KM augmentation. Porcine-derived acellular dermal matrix (PADM) has shown promise as a xenogeneic substitute for ADM, offering better resistance to contraction and improved esthetic integration, though its long-term outcomes require further research.^87,88^ Platelet-rich fibrin (PRF) membranes promote vascularization and healing but are limited by poor mechanical properties and rapid degradation.^89,90^ Silk fibroin matrices show potential in gingival tissue engineering but remain experimental with insufficient clinical validation.^91–93^

Overall, while FGG remains the gold standard for maximizing tissue augmentation, XCM and ADM provide viable alternatives that balance clinical and aesthetic outcomes.^94^ Despite the potential of these soft tissue substitutes, their efficacy and stability in various clinical scenarios still require robust, long-term evidence. Additionally, the usage of these substitutes significantly increases treatment costs, making clinical decision-making a balance between patient needs, esthetic demands, and financial considerations.

## Factors affecting the choice of PIKM augmentation surgeries

Although PIKM augmentation can be performed at almost any stage of implant treatment, the second-stage surgery is the most common timing for PIKM augmentation, as supported by substantial clinical evidence.

The decision-making process for choosing the appropriate PIKM augmentation surgery is influenced by several key factors, including the specific implant positions involved, the remaining width of the KM, and the extent of the required augmentation. A thorough preoperative assessment of the patient’s oral condition is essential for selecting the most suitable surgical approach. Additionally, other factors such as the technical sensitivity and patients’ preferences should also be taken into consideration. These factors determine not only the technical aspects of the surgery but also the potential for long-term success and the prevention of complications.

### Implant site

Implant site is the primary factor to consider. Due to the varying anatomical conditions and functional requirements of different implant sites, the applicable surgical techniques also differ accordingly.

In the aesthetic zone of the upper anterior region, the choice of PIKM augmentation is often influenced by considerations of gingival color aesthetics. While FGG is considered the gold standard, its potential to create a “patchy” appearance can negatively affect gingival aesthetics, making it less preferred. When soft tissue aesthetic outcomes need to be considered, a combination of SFGG with soft tissue substitutes is often the preferred choice, as it addresses both aesthetic and functional needs effectively.^69,95,96^

In the maxillary posterior region, due to the anatomical characteristics of the hard palate, the palatal side of the implant site is typically composed of KM. Therefore, when there is insufficient PIKM in the maxillary posterior region, it is usually manifested as a deficiency of buccal PIKM and a high frenulum attachment. In this case, based on the position of the MGJ, the depth of the vestibule, and the width of the KM of the adjacent teeth, the ARF or MARF is often chosen.^23,25^ These techniques can effectively use the palatal KM from the implant site to advance toward the buccal side, achieving the goal of increasing the PIKM and deepening the vestibule.^97^ Studies have shown that more than 3 mm of KM width can be achieved within one year postoperatively.^98^

The anatomical characteristics of the mandibular region differ from those of the maxilla. On the buccal side of the mandible, the presence of frenulum attachment often leads to insufficient width of KM. On the lingual side, there is the tongue and salivary glands, with more alveolar mucosa present, and the width of KM is typically inadequate. Additionally, in the mandibular posterior region, there is greater tension from the buccal musculature, and the insertion of muscle fibers makes the mucosa more prone to being stretched. If the mandibular region has insufficient PIKM requiring augmentation, the surgical technique chosen will mainly depend on the remaining width of the KM.

### The residual width of keratinized mucosa

The residual width of KM is another critical factor, particularly in the mandibular region, where it is a primary consideration. This is because the residual width of KM directly influences the applicability of certain surgical techniques. It is generally recommended that at least 4 mm of KM width should be required around dental implants, with a minimum of 2 mm on the buccal (labial) side and 2 mm on the lingual (palatal) side.^8,10^

As long as there is insufficient width of PIKM at the mandibular implant site, FGG remains the gold standard for augmentation, as it reliably increases both the width and thickness of KM.^99,100^ However, depending on the residual KM width, alternative surgical options may also be considered.

In cases where 3-4 mm of KM remains, ARF techniques can be employed effectively. ARF redistributes the existing keratinized tissue apically without requiring graft material, minimizing surgical morbidity and recovery time.

However, when the residual KM width is between 2 mm and 3 mm, the application of ARF is limited as the technique relies on adequate pre-existing tissue to achieve satisfactory outcomes.^101^ In such cases, grafting procedures such as FGG or SFGG are optional. For situations where donor site morbidity or esthetic concerns are priorities, SFGG combined with soft tissue substitutes can be considered.

In cases where less than 2 mm of PIKM remains (or when PIKM is completely absent), FGG are essential for providing the necessary keratinized tissue and functional stability. Compared to ARF or XCM, FGG ensure better long-term outcomes in peri-implant health and KM stability, especially in anatomically challenging regions like the posterior mandible.^102^

### The thickness of the recipient mucosa

The thickness of the recipient KM is another determinant, as thicker gingival biotypes are associated with better surgical outcomes, a reduced risk of gingival recession, and greater stability of the augmented tissue.^103^ Conversely, thin gingival biotypes may require pre-augmentation procedures to enhance soft tissue thickness before the primary surgical intervention.^104^ Thin PIKM is associated with several challenges, including an increased risk of mucosal recession and soft tissue instability, which can lead to both esthetic and functional complications.^105^ Additionally, thin KM correlates with greater tissue breakdown due to elevated levels of pro-inflammatory markers such as TNF-alpha, compromising peri-implant tissue health and stability.^106^ Patients with thin PIKM also report more pain and discomfort during oral hygiene practices, which affects their compliance with maintenance routines.^104^ Furthermore, thin PIKM reduces long-term peri-implant stability, increasing the likelihood of requiring surgical intervention to maintain implant health.^107^

Therefore, for patients with thin and insufficient PIKM, while ARF or soft tissue substitutes may be options for increasing the width of the PIKM, it is recommended to choose techniques that can also increase its thickness, such as FGG or MARF. Due to the presence of a certain thickness of connective tissue beneath the graft, these approaches enhance the thickness of the PIKM and improve the long-term prognosis of the implant.^108^

### Patient preferences

Patient preferences significantly influence the selection of surgical techniques for PIKM augmentation. Key factors include the patient’s tolerance for surgical trauma, economic considerations, and personal priorities such as aesthetics or recovery time. Aligning the surgical approach with patient preferences enhances satisfaction and adherence to postoperative care, highlighting the importance of thorough preoperative discussions and individualized treatment plans.^109^

Patients have varying levels of acceptance towards different surgical techniques. Procedures like FGG or SFGG require harvesting autogenous tissue from an additional donor site, which may provoke fear or hesitation in some patients.^110^ Although FGG does not rely on secondary healing to form new KM and allows for faster recipient site healing, donor site pain often significantly impacts the postoperative experience.^82^ The use of soft tissue substitutes eliminates the need to harvest autografts from donor sites and reduces pain at the recipient site.^100^ However, their predictability in widening and thickening PIKM is inferior to that of FGG. Studies have shown that minimally invasive methods, such as XCM, are often preferred by patients due to reduced pain and shorter recovery periods, despite potentially lower predictability compared to traditional FGG.^111^

Economic factors also play a crucial role, as less expensive techniques like connective tissue grafts may be more feasible for patients with limited financial resources, even though they require higher surgical precision.^82^

Additionally, patients who prioritize aesthetics often have specific expectations regarding the color and texture matching of augmented tissues. Techniques such as the use of xenogeneic collagen matrices have been shown to provide improved aesthetic outcomes due to their ability to closely match the appearance of natural tissue, making them appealing for patients with high aesthetic demands.^82^ Similarly, procedures combining SFGG with XCM have been reported to offer favorable aesthetic results while minimizing morbidity, further enhancing their acceptability for patients focused on aesthetics.^19^ Furthermore, living cellular constructs offer procedures with better tissue color matching, which yield more natural results despite being costlier and less widely available.^112^

### Other factors

Other factors can also significantly influence the choice of surgical methods for KM augmentation. However, the evidence supporting these factors remains insufficient, with much of the knowledge derived from expert experience.

Differences in surgical techniques among clinicians can impact the decision-making process, as some augmentation procedures are highly technique-sensitive.^113^ For less experienced surgeons, it is advisable to opt for simpler surgical approaches, such as the use of soft tissue substitutes, which not only improve the success rate but also reduce surgical time.^110^ Forcing the adoption of highly technically sensitive procedures without sufficient expertise often leads to unpredictable outcomes.

The height of residual alveolar ridge also influences the choice of technique. In cases of low alveolar bone height, vestibuloplasty may be less effective due to the shallow vestibular depth. Under these circumstances, surgical techniques that are less dependent on vestibular depth, such as FGG and MARF, are more suitable as they are less influenced by anatomical constraints. They help achieve predictable PIKM augmentation without being compromised by the limited ridge height or shallow vestibule.^32^

## Decision tree of PIKM augmentation at second-stage surgery

**Fig. 1:**
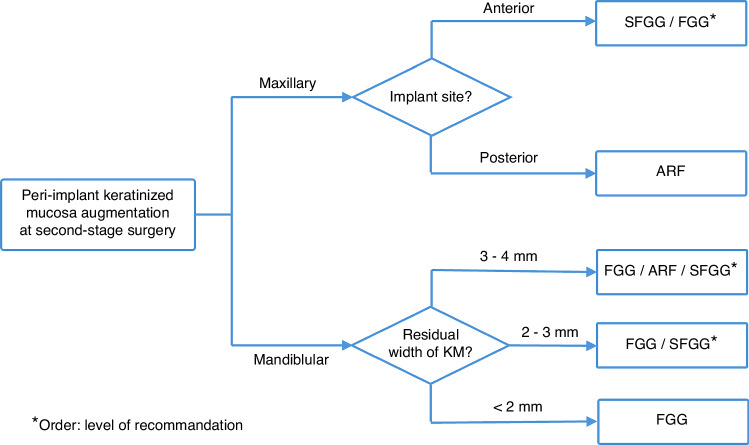
Decision tree of PIKM augmentation at second-stage surgery

Based on the techniques and factors discussed in the previous sections, we propose a decision tree to guide clinicians in selecting the most appropriate surgical approach for PIKM augmentation. This framework integrates considerations such as the residual width of KM and implant position, aiming to simplify clinical decision-making and enhance treatment outcomes. By addressing these variables systematically, the decision tree assists clinicians in tailoring surgical strategies to individual patient needs, ensuring predictable results and minimizing complications (Fig. 1).

- If PIKM in the maxillary anterior region (aesthetic zone) is insufficient, it is recommended to use the SFGG technique combined with soft tissue substitutes. This approach achieves a certain degree of increase in KM width while ensuring that the newly formed KM closely matches the original tissue in color and texture. In addition, FGG, as the gold standard, is also an alternative option and can be considered when aesthetics is not a primary concern.
- If PIKM in the maxillary posterior region is insufficient, the ARF technique is recommended, as the palatal KM in this region is usually sufficient for utilization.
- Once there is insufficient PIKM width in the mandible, FGG could be considered, especially in cases where the PIKM thickness is also inadequate.
- If the residual with of PIKM in the mandibular region between 3-4 mm, ARF, SFGG and FGG can be performed to increase the width of PIKM.
- If the residual width of PIKM in the mandibular region between 2-3 mm, SFGG or FGG can be performed. At this stage, the choice of surgical technique should be based on a comprehensive evaluation of factors such as patient preferences, aesthetic considerations, and the technique sensitivity of the procedure.
- If the residual width of PIKM in the mandibular region less than 2 mm, FGG is recommended before the second-stage surgery to augment the PIKM width.

## Conclusion and expectations

This consensus serves as a comprehensive guide for clinicians in selecting and performing PIKM augmentation procedures at second-stage surgery. It aims to standardize the surgical approach by offering a clear understanding of the various techniques and materials available for enhancing keratinized tissue around implants. By considering factors such as the timing of surgery, the remaining KM width, and the extent of augmentation required, clinicians can make more informed decisions to optimize patient outcomes. Additionally, the consensus emphasizes the importance of evaluating the technical sensitivity of each procedure, the costs of materials, and the unique needs of individual patients during decision-making process.

Future research in PIKM augmentation should focus on addressing the existing limitations in clinical evidence. Although there are high-quality clinical trials supporting certain techniques and materials, the available data remain limited in quantity, and the follow-up periods are often too short to fully assess long-term outcomes. Additionally, variations in reported outcomes across different studies, such as KM width and shrinkage rates, pose challenges to forming consistent clinical guidelines. This emphasizes the need for more comprehensive and standardized studies with extended follow-up durations to better understand the effectiveness and stability of current approaches. Therefore, future research should focus on prospective, multicenter randomized controlled trials with extended follow-up durations to evaluate the long-term effectiveness and stability of different augmentation techniques. Moreover, more standardized and objective measurement protocols, free from examiner bias, should be adopted to ensure greater accuracy and comparability in assessing KM thickness and stability across different patient populations. Standardized digital three-dimensional imaging could enhance the reliability of outcome assessments, allowing for better data synthesis and meta-analysis across different studies, ultimately leading to more robust and evidence-based conclusions.

Another area for improvement lies in achieving more natural aesthetic outcomes. Current techniques and materials, while effective in many cases, still face minor discrepancies in color and texture between regenerated and surrounding tissues, particularly in esthetically demanding regions. Future advancements should prioritize the development of new materials or techniques that can provide improved aesthetic integration, offering augmented tissues that closely resemble natural keratinized mucosa in both appearance and texture.

Lastly, the development of new materials for PIKM augmentation should focus on meeting specific clinical needs. These include achieving lower contraction rates to reduce shrinkage, ensuring greater predictability in attaining target KM width and thickness, and providing enhanced long-term stability. Furthermore, materials that offer multifunctionality, such as promoting tissue regeneration while maintaining wound protection, would significantly enhance clinical outcomes. As advancements in tissue engineering and regenerative medicine continue, innovative materials with these attributes have the potential to revolutionize KM augmentation, providing clinicians with reliable, predictable, and aesthetically superior options for long-term peri-implant success.
